# 

**Published:** 1896-04

**Authors:** 


					﻿Vol. XVI.
APRIL, 1896.
N0.4T
THE
pOMCEOPATHlC pHY^lClAJI,
A Monthly Journal of Homoeopathic Materia Medica
and Clinical Medicine.
“ He u a freeman whom truth maket free, and all are tlavet betide."
"Seek the Truth: come whence it may, coel what it will."
EDITED AND PUBLISHED BY
WALTER M. JAMES, M. D.
PHILADELPHIA:
JSTo. 1231 LOCUST STREET.
LONDON :•
ALFRED HEATH & CO., 8ole Agents fob Gbeat Britain,
114 Ebury Street, Eaton Square.
-Yearly Subscription, $2.SO, invariably in advance. Single Humbert, SB ctt.
Entered at the Philadelphia Poet-Offiee as Second-Claee Mail Matter.
				

